# Osilodrostat for Cyclic Cushing Disease

**DOI:** 10.1016/j.aed.2025.09.011

**Published:** 2025-09-30

**Authors:** Jane Rhyu, Run Yu

**Affiliations:** Division of Endocrinology, Diabetes, and Metabolism, University of California, Los Angeles (UCLA) David Geffen School of Medicine, Los Angeles, California

**Keywords:** osilodrostat, cyclic Cushing disease, cyclic Cushing syndrome

## Abstract

**Background/Objective:**

Cyclic Cushing syndrome is a rare subtype of Cushing syndrome with episodes of hypercortisolism, followed by spontaneous remission.

**Case Report:**

Our patient was a 68-year-old man who presented with his third cycle of cyclic Cushing disease with facial swelling, buffalo hump, fatigue, proximal muscle weakness, and lower extremity edema. Laboratory tests showed the following: (1) 24-hour urine free cortisol level, 12 030.3 mcg/d (normal, ≤60.0 mcg/d); (2) morning adrenocorticotropic hormone level, 464 pg/mL (normal, 6-59 pg/mL); (3) morning serum cortisol level, 91 mcg/dL (normal, 8-25 mcg/dL); and (4) potassium level, 3.3 mmol/L (normal, 3.6-5.3 mmol/L). Magnetic resonance imaging of the pituitary without/with contrast showed a partially empty sella. Prior inferior petrosal sinus sampling during the second cycle indicated a potential pituitary source of increased adrenocorticotropic hormone production, localized or draining to the right side. The patient was treated with osilodrostat with improvement in laboratory values and clinical symptoms by 2 to 3 weeks. After development of adrenal insufficiency, osilodrostat was rapidly titrated off by 2 months of treatment. Subsequently, laboratory findings after 8 days off osilodrostat confirmed clinical remission and reversibility of medication-induced adrenal insufficiency.

**Discussion:**

Because hypercortisolism is associated with mortality risk and comorbidities, timely management is a priority. If a surgical cure is not possible, a medication that treats hypercortisolism with rapid onset, reversible inhibition, and minimal side effects would be ideal to address the cyclicity.

**Conclusion:**

Our case is the first to our knowledge demonstrating osilodrostat’s use for native cyclic Cushing syndrome treatment and highlighted its reversibility and ability to preserve normal adrenal function.

**Highlights**

• Cyclic Cushing syndrome is a rare entity with significant comorbidities
• It is defined by at least 3 peaks of hypercortisolism and 2 troughs of eucortisolism
• Surgical cure is preferred, and medications are second-line
• Our case is the first showing successful treatment of native cyclic Cushing syndrome with osilodrostat
• Osilodrostat showed rapid onset/offset and reversible inhibition of steroidogenesis

**Clinical Relevance**

Osilodrostat is a new steroidogenesis inhibitor. Our case demonstrates the first successful treatment of native cyclic Cushing syndrome with osilodrostat, which showed rapid onset/offset, clinical safety, and reversible inhibition of steroidogenesis and medication-induced adrenal insufficiency. Osilodrostat’s preservation of underlying adrenal function is key when the cyclic Cushing episode spontaneously remits.

## Introduction

Cyclic Cushing syndrome (CCS) is a rare entity that represents a clinical challenge. It is defined by at least 3 peaks of biochemical hypercortisolism, which is clinically symptomatic in the majority, although rarely asymptomatic, and 2 troughs with normalized cortisol production that can last from days to years.^1^ The phenomenon can arise from any potential source of Cushing syndrome, including pituitary (54%), ectopic (26%), adrenal (11%), and unclassified (9%) sources.^1^ Intermittent hypercortisolism can also occur after pituitary surgery for Cushing disease.^2^

The cyclicity interferes with a straightforward diagnosis. It can lead to paradoxical results from biochemical testing and inferior petrosal sinus sampling,^3^ making determination of therapeutic outcomes more complicated.^3^ The goal of CCS management, as in all types of Cushing syndrome, is early diagnosis and intervention to reduce the length of hypercortisolism.^4^ A surgical cure is preferred because Cushing syndrome is associated with a fivefold increased standardized mortality risk.^4^ Cardiovascular, metabolic, bone, and cognitive comorbidities may persist despite remission and should be aggressively managed.^4^^,^^5^ For patients in whom surgical management is not possible or has not led to remission, medical therapy has a crucial role. We describe the first case to our knowledge of native CCS treated successfully with osilodrostat. A case of exogenous cyclic adrenocorticotropic hormone (ACTH)–dependent Cushing syndrome from pembrolizumab, with cyclicity attributed to the infusions, also demonstrated successful treatment with osilodrostat.^6^

## Case Report

The patient was a 68-year-old man with hypertension, hyperlipidemia, and rheumatoid arthritis with a history of cyclical episodes of weight gain and facial swelling, occurring spontaneously without steroid treatments. The initial episode occurred at the age of 62 years for 5 months and returned at the age of 64 years with facial swelling, buffalo hump, fatigue, proximal muscle weakness, sleep disturbances, and lower extremity edema. Laboratory tests showed the following (Table 1): (1) 24-hour urine free cortisol level, >245 mcg/d (normal, 11-84 mcg/d); (2) morning ACTH level, 528.0 pg/mL (normal, 7.2-63.3 pg/mL); and (3) morning serum cortisol level, 91.7 mcg/dL (confirmed on dilution; normal, 6.2-19.4 mcg/dL). Laboratory tests were also notable for a mildly low potassium level, low prolactin level, low testosterone level, and normal thyroid hormone, insulin-like growth factor-1, and dehydroepiandrosterone sulfate levels. Magnetic resonance imaging of the pituitary without/with contrast showed no sellar and suprasellar masses. Prior computed tomography of the abdomen/pelvis with contrast at the age of 62 years noted unremarkable adrenal glands. The patient was referred for inferior petrosal sinus sampling (Table 2), which indicated a potential pituitary source of increased ACTH production, localized or draining to the right side. The central-to-peripheral gradient values were ≥2 in the first prestimulation sample and ≥3 in all samples after providing 10 mcg of desmopressin (DDAVP). There was a >1.4/1 gradient between the right and left sides, suggesting a potential pituitary source draining to the right side (Table 2). The inferior petrosal sinuses were normal and of similar size. Cushing symptoms receded spontaneously in 5 months, and the patient did not follow up until recurrence at the age of 67 years.

**Table 1:** Laboratory Findings at Time of Onset of Cyclical Episodes

Laboratory test	Laboratory findings at the age of 64 y (second episode)	Laboratory findings at the age of 67 y (third episode)
24-h urine free cortisol level	>245 mcg/24 h (normal, 11-85 mcg/24 h)	12 030.3 mcg/d (normal, ≤60.0 mcg/d)
24-h urine creatinine level	1495 mg/24 h (normal, 1000-2000 mg/24 h)	1868 mg/d (normal, 800-2100 mg/d)
Morning ACTH level	528.0 pg/mL (normal, 7.2-63.3 pg/mL)	464 pg/mL (normal, 6-59 pg/mL),
Morning cortisol level	91.7 mcg/dL (normal, 6.2-19.4 mcg/dL)	91 mcg/dL (normal, 8-25 mcg/dL)
Thyroid-stimulating hormone level	0.452 mcIU/mL (normal, 0.450-4.500 mcIU/mL)	0.08 mcIU/mL (normal, 0.3-4.7 mcIU/mL)
Free thyroxine level	1.34 ng/dL (normal, 0.82-1.77 ng/dL)	1.30 ng/dL (normal, 0.8-1.7 ng/dL)
Prolactin level	<1.0 ng/mL (normal, 3.0-15.2 ng/mL)	8.05 ng/mL (normal, 3.5-19.4 ng/mL)
Insulin-like growth factor-1 level	148 ng/mL (normal, 64-240 ng/mL)	128 ng/mL (normal, 41-279 ng/mL)
Testosterone panel	Total, 66 ng/dL (11 am) (normal, 264-916 ng/dL) Free, 9.6 pg/mL (11 am) (normal, 6.6-18.1 pg/mL)	Total, 107 ng/dL (8:30 am) (normal, 300-720 ng/dL) Bioavailable, 61 ng/mL (8:30 am) (normal, 131-682 ng/mL)
Follicle-stimulating hormone level		3.6 mIU/mL (normal, 1.6-9 mIU/mL)
Luteinizing hormone level		1.6 mIU/mL (normal, 2-12 mIU/mL)
Dehydroepiandrosterone sulfate level	153 mcg/dL (normal, 48.9-344.2 mcg/dL)	
Potassium level	3.2 mmol/L (normal, 3.4-4.8 mmol/L)	3.3 mmol/L (normal, 3.6-5.3 mmol/L)
Creatinine level	0.92 mg/dL (normal, 0.7-1.2 mg/dL)	0.89 mg/dL (normal, 0.6-1.3 mg/dL)

Abbreviation: ACTH = adrenocorticotropic hormone.

**Table 2:** Inferior Petrosal Sinus Sampling

Timing of laboratory test in respective to DDAVP	Time	Right IPS ACTH level (normal, 6-59 pg/mL)	Left IPS ACTH level (normal, 6-59 pg/mL)	Inferior vena cava ACTH level (normal, 6-59 pg/mL)	Serum cortisol (normal, 8-25 mcg/dL)
Baseline 1	08:25 am	32	23	14	7
Baseline 2	08:27 am	19	16	13	7
Desmopressin (DDAVP)	08:30 am				
After 2 min	08:32 am	150	34	15	
After 5 min	08:35 am	123	32	18	
After 10 min	08:40 am	49	26	17	
After 15 min	08:45 am	124	31	17	
After 30 min	09:00 am	107	28	13	

Abbreviations: ACTH = adrenocorticotropic hormone; IPS = inferior petrosal sinus.

These results may indicate a pituitary source for increased ACTH production, localized or draining to the right side. There were central-to-peripheral gradient values of ≥2 (right IPS) in the first prestimulation sample and ≥3 in all 10-mcg postdesmopressin (DDAVP) samples. If due to an adenoma, it may drain into the right given the presence of a significant (greater than 1.4/1) gradient between the right and left. The inferior petrosal sinuses were of similar size and normal. These results should take into account the patient’s clinical scenario, and there are false positives and possible overlap with normal results.

**Fig. 1:**
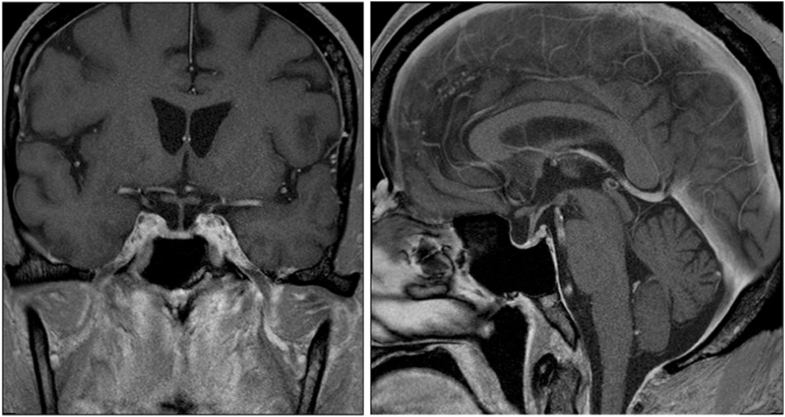
Magnetic resonance imaging (MRI) of the pituitary without/with contrast at the time of the third cyclical episode of Cushing disease. The MRI showed a partially empty sella with no evidence of a pituitary mass. Left, coronal view. Right, sagittal view.

During the third and most recent cycle of Cushing syndrome, laboratory tests after 1 month of symptom development showed the following (Table 1): (1) 24-hour urine free cortisol level, 12 030.3 mcg/d (normal, ≤60.0 mcg/d); (2) morning ACTH level, 464 pg/mL (normal, 6-59 pg/mL); (3) morning serum cortisol level, 91 mcg/dL (normal, 8-25 mcg/dL); (4) potassium level, 3.3 mmol/L (normal, 3.6-5.3 mmol/L); and (5) mild leukocytosis and erythrocytosis. Repeat magnetic resonance imaging of the pituitary without/with contrast showed a partially empty sella and no pituitary mass (Fig. 1).

**Fig. 2:**
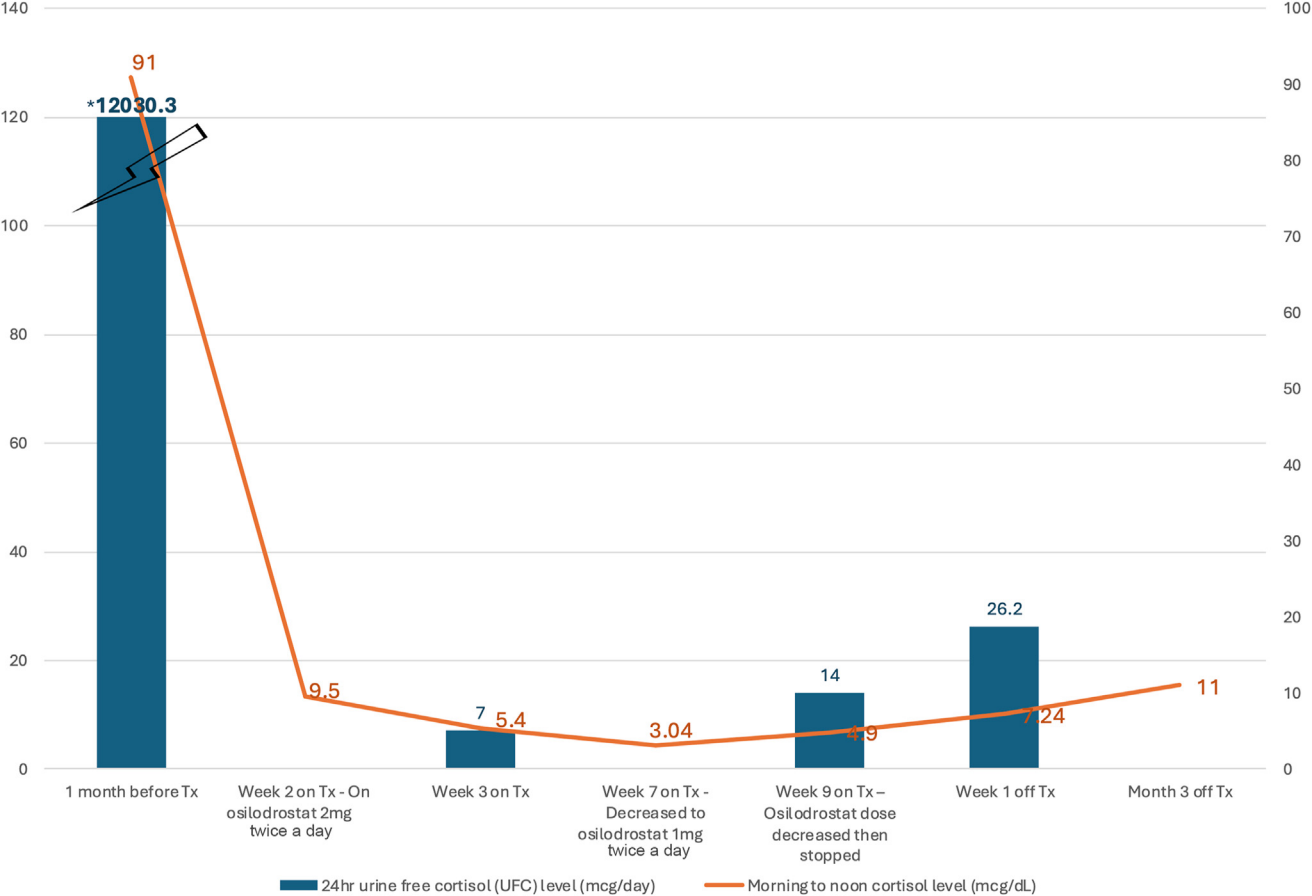
Trends of 24-hour urine cortisol levels and serum cortisol levels with osilodrostat treatment (Tx).

The patient was started on osilodrostat 2 mg twice daily. By week 2 of treatment, the morning cortisol level improved to 9.5 mcg/dL (8-25 mcg/dL), and the potassium level normalized, although facial and body swelling persisted. Significant improvement in symptoms and fatigue were noted by week 3 of treatment with the following laboratory findings: (1) morning ACTH level, 145 pg/mL (normal, 6-59 pg/mL); (2) morning serum cortisol level, 5.4 mcg/dL (8-25 mcg/dL); and (3) 24-hour urine free cortisol level, 7mcg/d (normal, 5-64 mcg/d). The osilodrostat dose was decreased to 1 mg twice daily and then 1 mg daily and stopped by 2 months of treatment after development of adrenal insufficiency (AI), which was confirmed on laboratory results (Table 3), along with corresponding symptoms of nausea, abdominal pain, low appetite, and fatigue. By that time, the facial and body swelling had also resolved. Potassium levels remained normal throughout treatment. After 8 days off osilodrostat, laboratory tests showed the following: (1) noon ACTH level, 67 pg/mL (normal, 6-59 pg/mL); (2) noon serum cortisol level, 7.24 mcg/dL (normal, 8-25 mcg/dL); and (3) 24-hour urine free cortisol level, 26.2 mcg/d (normal, ≤60.0 mcg/d). Nearly 3 months off osilodrostat, the patient had an 11 am ACTH level of 68.9 pg/mL (normal, 7.2-63.3 pg/mL) and 11 am serum cortisol level of 11.0 mcg/dL (6.2-19.4 mcg/dL). The clinical course is summarized in Table 3 and Figure 2. A DOTATATE-positron emission tomography scan was discussed, although the patient wished to reconsider in the future given clinical response.

**Table 3:** Laboratory Findings During Treatment With Osilodrostat

Laboratory test and treatment	1 month before Tx	Week 2 on Tx	Week 3 on Tx	Week 7 on Tx	Week 9 on Tx, Tx stopped	Week 1 off Tx	Month 3 off Tx
Treatment with osilodrostat	None	On 2 mg twice daily since week 0 of Tx	Advised to decrease to 1 mg twice daily but patient did not decrease dose	Decreased to 1 mg twice daily	Decreased to 1 mg daily after serum laboratory examination. Then discontinued Tx after 24-h UFC resulted in several days	None	None
ACTH level (pg/mL)	464		145	126	135	67	68.9
Cortisol level (mcg/dL)	91 8:32 am	9.5 7:04 am	5.4 7:11 am	3.04 11:56 am	4.9 11:26 am	7.24 12:14 pm	11 11:08 am
24-h UFC level (mcg/d)	12 030.3		7		14	26.2	

Abbreviations: ACTH = adrenocorticotropic hormone; Tx = treatment; UFC = urine free cortisol.

Normal reference ranges depending on assays: ACTH level, 6-59 pg/mL or 7.2-63.3 pg/mL; serum morning cortisol level, 8-25 mcg/dL or 6.2-19.4 mcg/dL; and 24-hour UFC level, ≤60.0 mcg/d or 5-64 mcg/d.

## Discussion

CCS is a rare subtype of Cushing and occurs in both ACTH-dependent and ACTH-independent cases.^3^^,^^7^ Cyclicity has been attributed to hypothalamic dysfunction exaggerating a normal variant of hormonal cyclicity, a dysregulated positive feedback mechanism followed by negative feedback, intratumoral bleeding, and ACTH secretion from neuroendocrine tumors (eg, carcinoid tumors and pheochromocytomas).^7^, ^8^, ^9^, ^10^

Potentially curative pituitary surgery or unilateral adrenalectomy are the treatments of choice.^4^ For example, cases of cyclic Cushing in primary pigmented nodular adrenocortical disease have demonstrated cure in some patients with unilateral adrenalectomy.^11^ In florid Cushing syndrome that is not amenable or responsive to other treatments, bilateral adrenalectomy could be lifesaving but risks significant comorbidities including Nelson syndrome.^4^^,^^12^ Pituitary radiotherapy/radiosurgery is a treatment option but risks progressive anterior pituitary dysfunction.^4^ Medical therapy can play an important role as a bridge to surgery or radiation, with recurrence, for poor surgical candidates, or when there is no identifiable source as in our patient.^13^ CCS, moreover, has a higher recurrence rate (63%) and lower remission rate (25%) than classic Cushing syndrome.^8^

Medical treatments of CCS include steroidogenesis inhibitors (ketoconazole, levoketoconazole, metyrapone, and osilodrostat), adrenolytic agents (mitotane), glucocorticoid receptor blockers (mifepristone), and pituitary tumor-directed agents (pasireotide, cabergoline, and temozolomide).^8^^,^^14^^,^^15^ Treatment goal is normalization of 24-hour urine cortisol levels and morning serum cortisol levels, although block-and-replace regimens occasionally are used.^13^^,^^14^ A block-and-replace regimen with osilodrostat and dexamethasone was used in the case of exogenous cyclic Cushing from pembrolizumab, given need for the immunotherapy^6^; however, this regimen would hinder assessment of remission in native cyclic Cushing.

As our patient had cyclic Cushing disease, pituitary tumor-directed medications could be used for treatment. Pasireotide and cabergoline, however, are limited by a significant percentage of nonresponders, along with the risk of hyperglycemia for pasireotide.^15^ We considered mifepristone, which is a competitive antagonist at the glucocorticoid receptor and progesterone receptor; however, mifepristone is limited by the inability to directly monitor cortisol response on laboratory examinations, in addition to the risk of AI and mineralocorticoid side effects with overtreatment.^16^

Steroidogenesis inhibitors block 1 or more enzymes in the production of cortisol, with potential risk of AI. The new steroidogenesis inhibitor osilodrostat, such as metyrapone, selectively inhibits CYP11B1 and CYP11B2, which are involved in the final steps of cortisol and aldosterone synthesis, respectively.^13^^,^^14^ Ketoconazole and levoketoconazole, on the other hand, block most enzymes in the adrenal steroidogenesis pathway, including CYP11B1 and CYP11B2, and are limited by their inhibition of CYP7A (with associated hepatotoxicity) and strong inhibition of cytochrome p450 CYP3A4 (leading to many drug-drug interactions, decreased testosterone production, and QTc prolongation).^14^

Osilodrostat and metyrapone do not affect CYP7A and less potently inhibit CYP3A4.^13^ However, they can lead to increased deoxycorticosterone levels, with associated risks of hypokalemia, hypertension, and edema, and increased androgen production (with metyrapone thus being considered second-line in women).^13^^,^^14^^,^^17^

Osilodrostat, compared with metyrapone and ketoconazole, has a higher potency in CYP11B1 and CYP11B2 inhibition and a longer half-life, with stronger effects in lowering cortisol levels, allowance of less frequent (twice daily) dosing, and possibly less side effects.^13^^,^^14^^,^^17^^,^^18^ Compared with metyrapone, studies have suggested osilodrostat leads to a lesser increase in 11-deoxycortisol levels and less hyperandrogenic effects.^13^^,^^14^ Osilodrostat is also rapidly absorbed with sustained efficacy up to 6.7 years.^17^^,^^18^ Although rare cases of prolonged AI following discontinuation exist, osilodrostat (similar to other steroidogenesis inhibitors) is generally considered a reversible inhibitor.^19^ Reversible inhibition of cortisol synthesis is particularly appealing to treatment of CCS because patients will not suffer from prolonged AI after episodes subside.

We, thus, considered osilodrostat an attractive treatment of CCS. In our patient, osilodrostat was efficacious and well tolerated, consistent with the literature,^17^ with clinical effects within 2 to 3 weeks without significant mineralocorticoid side effects. Differentiation of AI as a side effect of osilodrostat or from remission of the cyclical episode is crucial. Our patient was carefully tapered off osilodrostat after developing AI, and reversal of AI and osilodrostat inhibition were clearly demonstrated after 8 days off osilodrostat. Off treatment, the patient demonstrated neither prolonged AI nor clinical hypercortisolism, confirming remission of cyclic Cushing.

## Conclusion

We present the first case to our knowledge demonstrating successful treatment of native CCS with osilodrostat. Osilodrostat showed rapid and safe control of hypercortisolism and importantly exhibited quick reversible inhibition of steroidogenesis upon discontinuation, a virtue in CCS management.

## Disclosure

The authors have no conflicts of interest to disclose.
